# Protein Replacement Therapy Partially Corrects the Cholesterol-Storage Phenotype in a Mouse Model of Niemann-Pick Type C2 Disease

**DOI:** 10.1371/journal.pone.0027287

**Published:** 2011-11-03

**Authors:** Gitte Krogh Nielsen, Frederik Dagnaes-Hansen, Ida Elisabeth Holm, Steve Meaney, Derek Symula, Niels Trolle Andersen, Christian Würtz Heegaard

**Affiliations:** 1 Department of Molecular Biology, Aarhus University, Aarhus, Denmark; 2 Department of Medical Microbiology and Immunology, Aarhus University, Aarhus, Denmark; 3 Experimental Neuropathology Laboratory, Department of Pathology, Randers Hospital and Clinical Institute, Aarhus University, Aarhus, Denmark; 4 School of Biological Sciences, Dublin Institute of Technology, Dublin, Ireland; 5 Wadsworth Center, Albany, New York, United States of America; 6 Department of Biostatistics, Aarhus University, Aarhus, Denmark; Virginia Commonwealth University, United States of America

## Abstract

Niemann-Pick type C2 (NPC2) disease is a fatal autosomal recessive neurovisceral degenerative disorder characterized by late endosomal-lysosomal sequestration of low-density lipoprotein derived cholesterol. The breach in intracellular cholesterol homeostasis is caused by deficiency of functional NPC2, a soluble sterol binding protein targeted to the lysosomes by binding the mannose-6-phosphate receptor. As currently there is no effective treatment for the disorder, we have investigated the efficacy of NPC2 replacement therapy in a murine gene-trap model of NPC2-disease generated on the 129P2/OlaHsd genetic background. NPC2 was purified from bovine milk and its functional competence assured in NPC2-deficient fibroblasts using the specific cholesterol fluorescent probe filipin. For evaluation of phenotype correction *in vivo*, three-week-old *NPC2* ^−/−^ mice received two weekly intravenous injections of 5 mg/kg NPC2 until trial termination 66 days later. Whereas the saline treated *NPC2* ^−/−^ mice exhibited massive visceral cholesterol storage as compared to their wild-type littermates, administration of NPC2 caused a marked reduction in cholesterol build up. The histological findings, indicating an amelioration of the disease pathology in liver, spleen, and lungs, corroborated the biochemical results. Little or no difference in the overall cholesterol levels was observed in the kidneys, blood, cerebral cortex and hippocampus when comparing *NPC2* ^−/−^ and wild type mice. However, cerebellum cholesterol was increased about two fold in *NPC2* ^−/−^ mice compared with wild-type littermates. Weight gain performance was slightly improved as a result of the NPC2 treatment but significant motor coordination deficits were still observed. Accordingly, ultrastructural cerebellar abnormalities were detected in both saline treated and NPC2 treated *NPC2* ^−/−^ animals 87 days post partum. Our data indicate that protein replacement may be a beneficial therapeutic approach in the treatment of the visceral manifestations in NPC2 disease and further suggest that neurodegeneration is not secondary to visceral dysfunction.

## Introduction

Niemann-Pick type C (NPC) disease is a rare autosomal recessive, lysosomal storage disorder characterized by intracellular accumulation of cholesterol and other lipids throughout the body [1], [2]. The etiology of the disease resides in mutations that inactivate either of two proteins, NPC1 (95% of cases) or NPC2 (5% of cases) [3], [4]. These two proteins are structurally distinct - NPC1 is a large transmembrane protein situated in late endosomes and NPC2 is a small soluble sterol-binding glycoprotein (see [5] for a recent review). While the precise function of these proteins is not completely understood, the resemblance of both the clinical manifestations and biochemical abnormalities suggests that they function in concert to facilitate the egress of lipoprotein-derived cholesterol from the endo-lysosomal system [6], [7], [8]. The clinical spectrum of NPC disease is relatively broad, ranging from a rapidly fatal neonatal disorder to a chronic neurodegenerative disease with adult onset. Apart from the approximately 10% of the patients who die before 6 months of age due to liver- and respiratory failure, most patients ultimately develop progressive neurological dysfunction, which typically is preceded by varying degrees of hepatosplenomegaly. Typical neurological symptoms include vertical supranuclear gaze palsy, cerebellar ataxia, dysarthria, dysphagia, and progressive dementia [1], [9], [10].

To date there is no effective therapy for NPC disease, although encouraging results have emerged from recent preclinical studies using the sterol chelator 2-hydroxypropyl-β-cyclodextrin (CD) and the glucosylceramide synthase inhibitor miglustat [11], [12], [13], [14]. Bone marrow transplantation has been attempted in two patients diagnosed with NPC2 disease. Unfortunately, the first patient died two months after the procedure from an adenovirus infection [15] and a long-term follow up is required before the efficacy of the second bone marrow transplant can be ascertained [16].

Enzyme replacement therapy (ERT) has previously been used with variable success in a number of lysosomal storage disorders, including Gaucher disease, Mucopolysaccharidosis types I, II and VI, Fabry disease, and Pompe disease [17], [18], [19]. ERT relies on the capacity of cells to endocytose exogenously supplied lysosomal enzymes and transfer them to the lysosomes [20]. This is usually done via the mannose-6-phosphate (M6P)-receptors and the mannose receptor. In the initial study elucidating the molecular basis for NPC2 disease, conditioned medium from Chinese hamster ovary cells secreting recombinant NPC2 was able to correct the cholesterol-laden phenotype of cultured NPC2 deficient fibroblasts in a M6P inhibitable manner [4]. Thus, the NPC2 protein appears a suitable candidate for replacement therapy and may potentially provide the means to impede and correct the progression of the disease.

NPC2 is a highly conserved small glycoprotein (approx. 16 KDa), which is present in all mammalian tissues examined [4], [21]. In common with many other lysosomal proteins, it is present in low quantities and cannot be directly purified from tissue in significant amounts. However, NPC2 is also found in secretory fluids such as epididymal fluid [22], milk [21], bile and plasma [23]. Our laboratory originally purified and structurally analyzed milk derived bovine NPC2, called EPV20 [21]. The protein is relatively abundant in bovine milk, which facilitates isolation of quantities sufficient for crystallographic studies [24], [25]. Mature milk derived bovine NPC2 contain 130 amino acid residues, the same length as the deduced amino acid sequence of its murine homologue. By comparison, the predicted primary sequence of human NPC2 is extended with additional two amino acid residues. The amino acid sequence identity between the milk derived bovine NPC2 and human NPC2 is 79% and between bovine NPC2 and murine 75%. In addition, the spatial distribution of the six cysteine residues known to be involved in intra-chain disulfide bonds in the bovine protein is the same in the human and murine counterparts. Also, the secreted bovine protein was capable of binding to the cation*-*independent M6P/IGF2-receptor by a mechanism which could be inhibited by M6P (own unpublished data) and the cholesterol binding domains essential for ligand transport are preserve in the milk derived protein [21], [24]. Furthermore, the purified protein does not carry the mutations exhibited in the NPC2 patient population. Taken together, the structural comparison suggests that milk-borne NPC2 potentially can complement the loss of endogenous NPC2. The availability of an abundant source of NPC2 protein and a murine NPC2*-*hypomorph animal model have made it possible, for the first time, to test if NPC2 replacement therapy is a potential therapeutic strategy for NPC2 disease. The question was explored by comparison of physical, biochemical and histological alterations in NPC2 treated *NPC2* ^−/−^ mice, saline mock-treated *NPC2* ^−/−^ mice, and age-matched wild type littermates. Intravenous injections of bovine NPC2 lead to a marked systemic improvement in the *NPC2* ^−/−^ mice, although the chosen intervention was unable to prevent the progressive neurodegeneration associated with the disease. The results highlight an effective novel approach for treatment of the visceral manifestations in NPC2 disease.

## Materials and Methods

### Purification and characterization of NPC2

Naturally occurring NPC2 was purified from bovine skim milk by selective acid and ammonium sulfate precipitation followed by a two-step ion-exchange procedure as previously described [21]. The purity and identity of the purified protein was determined by SDS-polyacrylamide gel electrophoresis (SDS-PAGE) in 10–20% gradient gels and N-terminal amino acid sequence analysis (ABI 477A/120A, Applied Biosystems Inc.), respectively. Protein concentrations were determined by acid hydrolysis followed by quantitative amino acid analysis based on o-phtaldialdehyde derivation.

### Endotoxin removal

Purified NPC2 was dialyzed into endotoxin-free Dulbecco's Phosphate Buffered Saline, pH 7.4 (D-PBS) (Gibco). The retentate was applied to a DEAE-sepharose column equilibrated in D-PBS, pH 7.4. The quantity of lipopolysaccharide (endotoxin) present in the flow through NPC2 preparation was determined using a *Limulus amebocyte* lysate assay (LAL-assay, Lonza, Inc.) according to the manufacturer instructions. The purified NPC2 was sterile-filtered and stored frozen at –20°C until use.

### Cell lines and primary embryonic cells

Normal human skin fibroblasts (GM08680) were obtained from American Type Culture Collection (Rockville, MD, USA). The NPC2-null mutant human skin fibroblast cell line, NPC2^G58T^ (NIH 99.04) was a kind gift from Anthony H. Fensom (Paediatric Research Unit, United School of Guy's Hospital, London). Primary mouse embryonic fibroblasts were isolated from 13 days old fetuses bearing *NPC2^+/+^* and *NPC2* ^−/−^ (129P2/OlaHsd-*NPC* ^Gt(LST105)BygNya^) genotypes. The head and internal organs were removed, and the torso was minced and dispersed in 0.1% trypsin for 10 min at 37°C. All cells were plated in DMEM containing 10% Fetal Calf Serum (FBS) and 1% penicillin/streptomycin and routinely maintained at 5% CO_2_ and 37°C. A small piece of tissue from each embryo was used to determine the genotype as described below.

### Filipin fluorescence staining of free cholesterol

Fibroblasts were seeded onto glass cover slips in 24*-*well tissue culture microplates in DMEM medium containing 10% FCS and grown to ∼80% confluence (8×10^5^ cells/well) and then incubated for 48 hours with 10 µg/ml NPC2 in a 0.5 ml working volume. To examine the cholesterol load the fibroblasts were fixed in 10% phosphate buffered formalin, pH 7.2 for 30 min, washed three times in D-PBS containing calcium and magnesium and then incubated for one hour with Filipin III (10 µg/ml) in PBS∶DMSO (50∶1). Subsequently, the fibroblasts were washed three times in D-PBS. Coverslips were examined with a Zeiss Axioplan2 imaging fluorescence microscope equipped with a Axiocam digital camera/ACT-1 software. Visualization of filipin images was performed using 360/40 nm excitation and 465/30 emmision filters.

### Animals

129P2/OlaHsd-*NPC* ^Gt(LST105)BygNya^ mice holding the LST105 mutation were used in the present study. The LST105 mutation yields a fusion protein that includes the first 27 amino acids of the NPC2 protein (encoded by the first exon of the *NPC2* gene), including the 19 amino acids signal sequence. Mice heterozygous for the LST105 gene trap mutation were interbred to obtain siblings homozygous for the gene trap (*NPC2* ^−/−^) and wild type (*NPC2* ^+/+^) alleles. The *NPC2* ^−/−^ and *NPC2* ^+/+^ littermates were housed in standard type II and type III plastic cages with filter tops (Technipast, Italy) at an ambient temperature of 20°C and with altering 12 hour periods of dark and light. The animals were given ad libitum access to water and standard low-cholesterol (0.02% w/w) chow (Altromin 1314, Brogaarden A/S, Gentofte). The experimental protocol was approved by The Danish Experimental Animal Inspectorate (Permit numbers 561-1407 and 562-25).

### Genotyping

All mice were genotyped at postnatal day 16 (P16). DNA from ear tags was prepared using the N-extract tissue kit (Sigma-Aldrich; cat. no. XNATR). Polymerase chain reactions (PCR's) were performed using the two set of primer pairs (NPC2-mutant: forward primer 5′-CCA GGC AGC ACG GAT GTC-3′ and reverse primer 5′-GCC AGG GTT TTC CCA GTC A-3′) and (NPC2-wt: forward primer 5′-TGT GGC TCA GTG GCT TAG G-3′ and reverse primer 5′-CCA GGA AGG GAT TTC ACA CA-3′). The PCR-products were run on a 1.5% agarose gel in 1xTBE buffer and stained in Midori Green (Nippon Genetics). Analysis of the gels was performed on a Typhoon scanner (GE Healthcare).

### Measurement of murine immune response to NPC2

For an initial evaluation of the septic potential and antibody response to infusion of the bovine NPC2 protein preparation, eight *NPC2* ^+/+^ mice were equally divided in two groups and challenged weekly with 5 mg/kg NPC2 injected intraperitoneally (i.p.) or intravenously (i.v.), respectively. Blood was drawn and serum isolated before treatment start (pre-immune) and ones a week for five weeks. The mice were anesthetized with isoflurane before the blood was sampled from the retro-orbital sinus using a fine-walled Pasteur pipette (o.d. of 1–2 mm). Anti-NPC2 IgG positive serum was produced by subcutaneous injections of Freund Complete Adjuvants mixed with NPC2 protein. The injection was repeated three weeks later and serum was prepared 6 weeks after the last immunization. A relative quantification of anti-NPC2 IgG, was performed using a time-resolved immune fluorometric assays (TRIFMA); 100 µl purified NPC2 (1 µg/ml) in D-PBS, pH 7.4 was incubated for 18 hours in 96-well microtiter plates at room temperature (RT). The wells were washed three times in washing buffer 1 (WB1: TBS, 0.05% Tween 20) and then blocked for one hour with WB1 at RT. 100 µl purified mouse plasma diluted 1∶1000, 1∶3000, and 1∶9000 in WB1 were added to the NPC2 coated wells and incubated at RT for one hour. After incubation, the WB1 washing was repeated, followed by one hour incubation at RT with 100 µl of biotin-conjugated rabbit anti-mouse IgG (1 µg/ml; DAKO). The primary antibody was removed by washing in WB1 and rinsing in washing buffer 2 (WB2: TBS, 0.05% Tween 20, 25 µM EDTA). Next Eu^3+^-conjugated streptavidin (Sigma Aldrich) diluted in WB2 was added to the wells and incubated for one hour at RT. The wells were washed three times in WB2 and bound europium was detected on a Delfiar fluorometer (Perkin Elmer). Anti-NPC2 IgG responses were calculated by subtracting the mean background signal (buffer only) from the mean anti-NPC2 IgG signal. Using the same technique *NPC2^−/−^*- and wild type mice enrolled in the NPC2 replacement trial were tested for anti-NPC2 activity just before and weeks 4, 8, and 10 after commencing the NPC2 treatment trial.

### NPC2 replacement therapy procedure

To study the long-term effect of NPC2 replacement therapy on the disease phenotype we established a colony of NPC2 deficient (*NPC2^−/−^*) and wild type (*NPC2* ^+/+^) siblings from mating *NPC2^+/−^* mice. The offspring were divided into three groups, each containing ten animals. In the first group, three-week-old *NPC2* ^−/−^ mice were treated twice/week with a tail vein injection of 5 mg/kg. In the second group, *NPC2* ^−/−^ control mice received saline solution. The third group comprised age-matched saline treated *NPC2* ^+/+^ littermates. All groups were injected with the same volume and frequency. Hypersensitivity reactions and mortality were absent throughout the entire treatment period. Other possible side effects were monitored by daily inspection of cage behaviour, food and water consumption. Body weights were measured once weekly and serum samples were collected every other week just prior to treatment. At P87, the mice were euthanatized, and organs (liver, spleen, lungs, kidneys, and brain) were weighted and secured for biochemical and histological analyses. Tissue sections used for biochemical analyses were snap frozen using liquid nitrogen and stored at −80°C, whereas sections for histological and immunohistochemical examination were immersion fixed in 4% phosphate buffered paraformaldehyde for at least 48 hours.

### Histological and Immunohistochemical staining

Paraformaldehyde fixed tissues from three mice in each of the three experimental groups (NPC2 treated *NPC2^−/−^* mice, saline treated *NPC2^−/−^* mice, and saline treated wild type mice) were placed in PBS overnight and then dehydrated in ethanol and xylene, and embedded in paraffin. For routine histology, 5 µm-thick paraffin sections were rehydrated and stained with haematoxylin and eosin (H&E), periodic acid-Schiff (PAS), or Masson Trichrome stain. Before histological immunostaining endogenous peroxidase activity was eliminated by incubation with 3% hydrogen peroxide for 10 minutes and the sections were then washed in running tap water for 10 minutes. Following heat-mediated antigen retrieval in Tris-buffer, pH 8.5, sections were rinsed in PBS-buffer, pH 7.6 and incubated for 30 minutes with Rat anti-mouse F4/80 antibody (1∶100) (Invitrogen). The primary antibody was removed and the slices washed two times for five minutes in PBS, pH 7.6, before incubation for 30 minutes with HRP-conjugated anti-rat immunoglobulin (1∶100) (P0450, DAKO). After repeated rinsing in PBS the sections were stained with 3.3-diaminobenzidine tetrahydrochloride (DAB) substrate and counterstained with haematoxylin. The sections were washed in running tap water, dehydrated and mounted. Brightfield images were obtained using an upright Leica microscope (DM2500) equipped with a Leica digital camera (DGC320). An experimenter blinded to genotype and treatment status undertook all procedures and post-staining image analyses.

### Cholesterol extraction and quantification

Tissue samples were obtained from six mice in each of the three experimental groups and total cholesterol was extracted by homogenization in chloroform/methanol (2∶1) according to Folch [26]. Briefly, 10 mg of tissue was homogenized in two ml D-PBS, pH 7.4, containing a protease inhibitor cocktail with EDTA (Roche) and 1 mM butylated hydroxytoluene (Sigma Aldrich), using a tissue lyzer II apparatus (Qiagen). 0.6 mL of the homogenate was extracted with three volumes of chloroform/methanol (2∶1, v/v). The mixture was vortexed for one min and centrifuged 10 min at 1000 x *g*. The extraction of the upper phase (aqueous phase) was repeated five times and the extract was concentrated under a stream of nitrogen and re-dissolved with 0.1 mL 2-propanol. Total cholesterol was measured by an enzymatic, colorimetric, endpoint method according to the manufacturer instructions (Randox). To determine the reliability of the colometric method used, each tissue homogenate was divided in two and each half was analyzed separately. In analyzing variance, the two samples obtained from each mouse were averaged. No difference in cholesterol levels per mg wet tissue was observed between males and females organs. Therefore, the means of total cholesterol in all tissues are generated based on data from both genders.

### Statistical data analysis

Quantitative data were statistically evaluated with one-way analysis of variance (ANOVA) and analyses were performed on ln-transformed data. Multiple comparison ad hoc tests were performed by pair wise comparisons. In order to interpret results in percentage terms, estimates of differences between groups were transformed by exp and 95% confidence intervals (CI) were calculated for these relative differences. To compare the relative weight change in each of the treatment groups, the relative weight change from day 70 to 87 was calculated for each animal and the saline-treated *NPC2* ^+/+^, saline-treated *NPC2^−/−^* and the NPC2-treated mice were compared by ANOVA. Homogeneity of variance was tested using Bartletts test. The statistical software used was STATA version 10 and the significance level was set to 5%.

## Results

### Preparation and *in vitro* activity validation of milk derived NPC2

**Figure 1:**
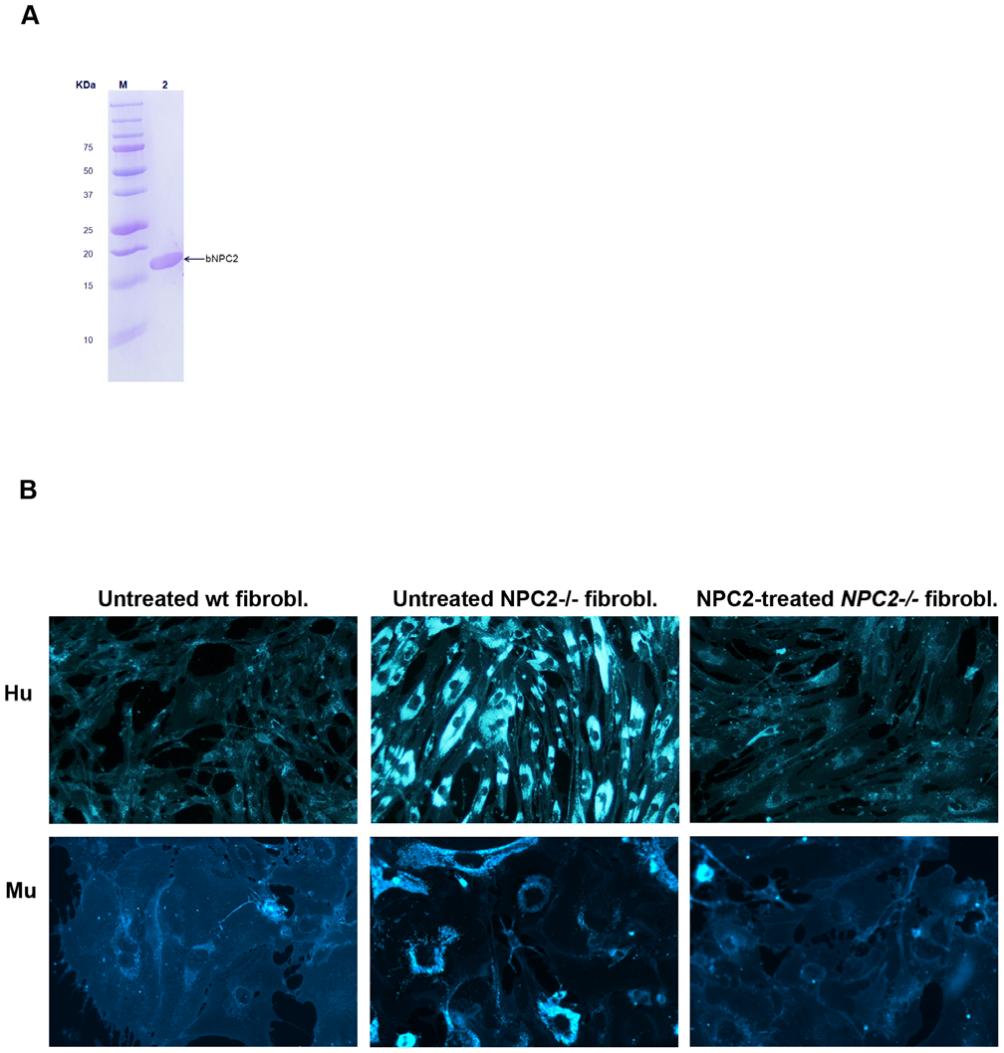
Effect of purified bovine NPC2 on cholesterol accumulation in wild type and *NPC2^−/−^* fibroblasts. (**A**) SDS-PAGE of 5 µg purified NPC2 resolved in a 10–20% gradient gel under nonreducing conditions and stained with Coomassie Brilliant Blue (*lane 1*). Molecular mass markers are shown on the left (*lane M*). (**B**) Upper row human- (Hu) and lower row murine (Mu) fibroblasts cultivated for 48 hours in complete medium (DMEM + 10% FBS). Wild type fibroblasts (*left panels*), *NPC2^-/-^* fibroblasts (*middle panels*), *NPC2^-/-^* fibroblasts supplemented with 600 nM NPC2 (*right panels*). Cells were fixed with 10% phosphate buffered formalin, pH 7.4 and stained with Filipin III and visualized using fluorescence microscope.

Endogenous NPC2 was purified from bovine milk by sequential anion- and cation- exchange chromatography as previously described [21]. An additional anion exchange chromatography step was performed to selectively remove or decrease contaminating endotoxins from the eluted protein preparation. Upon electrophoresis in SDS–polyacrylamide gels, the resulting NPC2 migrated as a single band with the expected mobility (apparent molecular mass of ∼20 kDa) (Fig. 1 A). The identity and purity (>95%) of the NPC2 preparation was further confirmed by N-terminal Edmann sequencing. Amino acid analysis and Limulus test revealed that the average yield of purified NPC2 was 2 milligrams per litre of milk, holding less than 5 endotoxin units per mg protein (data not shown). To confirm the functional competence of the milk-derived NPC2 preparation we used filipin fluorescence staining of free cholesterol in cultivated human and murine fibroblasts. Intense labelling of punctuated vesicular structures throughout the cytoplasm was observed in human skin fibroblasts with a null mutation (Glu20stop) in the *NPC2* gene as well as in the primary culture of murine embryonic *NPC2^−/−^* fibroblasts developed by mating NPC2-targeted heterozygous (Fig. 1 B, *middle panels*). Addition of 600 nM purified NPC2 in the culture medium corrected the cholesterol storage phenotype in both the human and murine *NPC2^−/−^* fibroblasts, as evidenced by the marked decrease in filipin fluorescence to a level observed for the wild type cells (Fig. 1 B, *right and left panels*). A ten-fold reduction in the NPC2 concentration, equivalent to ≈375×10^5^ molecules of NPC2 added per cell, did not alter this outcome (not shown). The cross-species correction of defective recipient cells means that the bovine NPC2 preparations fulfil the first criteria for a successful introduction of the protein in the murine NPC2 disease model.

### Assessment of humoral immune response to bovine NPC2 in mice

**Figure 2:**
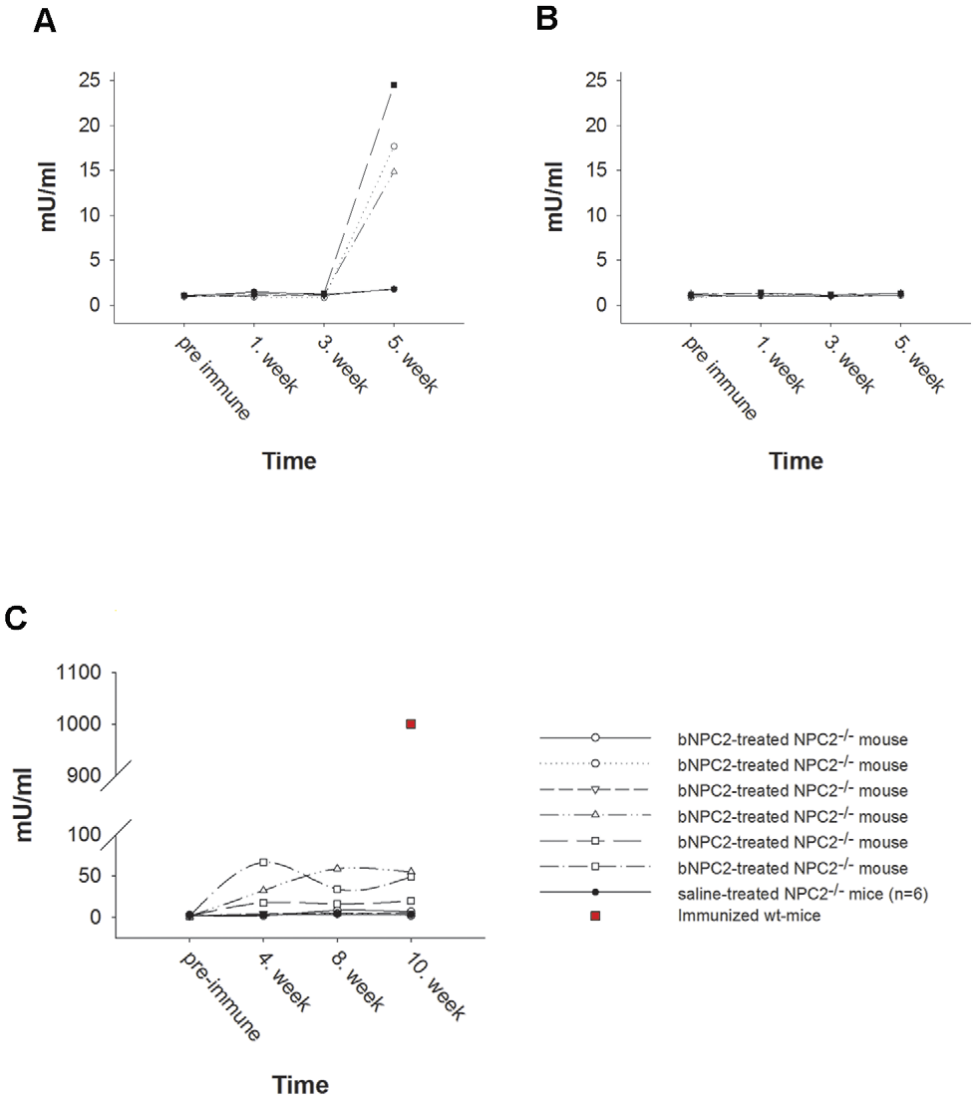
Immune response to NPC2 in 129P2 wild type- and *NPC2^−/−^* mice. NPC2 coated microtiter wells were incubated with serial dilutions of immunized serum as indicated. Bound antibodies were detected by TRIFMA as described in Materials and Methods. An arbitrary concentration of anti-NPC2 antibodies was set to 1000 mU/ml in positive control serum prepared by subcutaneously injections of NPC2 with Freund's complete adjuvant as immune potentiator. Sera from saline treated healthy mice were similarly tested and served as negative controls. (**A**) Anti-NPC2 antibody concentration in intraperitoneal injected wild type mice, (**B**) Anti-NPC2 antibody concentration in intravenous injected wild type mice, and (**C**) Anti-NPC2 antibody concentration in intravenous NPC2 treated *NPC2^−/−^* mice. Data represents mean values of triplicate wells.

Monitoring of antibody production during protein replacement therapy is an important consideration as high-titer neutralizing antibody translates into a lack of clinical response and affect the safety of the therapy [27]. Thus, as fluorometry based immunoassay was designed for the detection of anti-NPC2 IgG in mouse serum. During the five-week evaluation period, no clinical signs consistent with a hypersensitivity reaction were observed resulting from weekly injections of saline or 5 mg/kg NPC2. In some animals, however, we did see a minor boost in NPC2 specific antibodies caused by intraperitoneal injection (Fig. 2A). As intravenous tail vein injections revealed levels of anti-NPC2 IgG similar to the baseline levels observed in saline-treated mice (Fig. 2B), we chose this route of administration to study the long-term effect of NPC2 replacement therapy. During the ten-week replacement therapy trial (performed as described below), no hypersensitivity reactions were observed in any of the enrolled mice. However, Anti-NPC2 IgG in variable amounts was detectable in the some of the NPC2 treated mice, but the titers were 20 to 200-fold lower than those observed in serum from NPC2 immunized mice (Fig. 2C).

### NPC replacement therapy reduces weight loss in *NPC2^-/-^* mice

**Figure 3:**
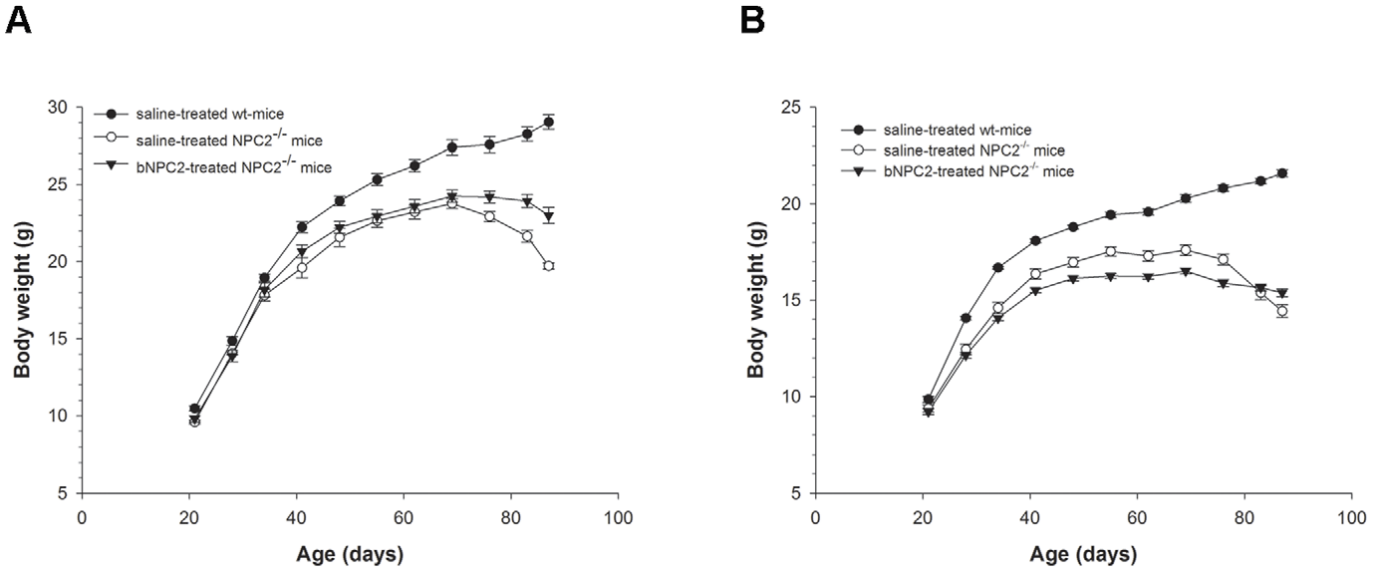
Effect of NPC2 treatment on animal body weight. (**A**) Males (n = 4) and (**B**) females (n = 6), respectively, of saline-treated wild type mice (•), saline-treated *NPC2^−/−^* mice (○), and NPC2 treated *NPC2^−/−^* mice (▾). The mice were weighed weekly from P21 to P87. Each animal was injected twice weekly with saline or NPC2 (5 mg/kg). Values are means ± SEM.

In order to study the efficacy of NPC2 replacement therapy, offspring of heterozygote NPC2 mutant breeding pairs were divided into three groups, each containing ten animals. In the first and second group, three-week-old *NPC2* ^−/−^ mice received NPC2 (5 mg/kg) or saline, respectively, by tail vein injection twice weekly for 66 days. The third group comprised age-matched saline treated *NPC2* ^+/+^ littermates, injected with the same volume and frequency. A reported common feature of *NPC1* gene ablated and NPC2 hypomorphic mice is rapid weight loss at some point during the disease progression [8]. In the NPC2 deficient mouse model used in this study, weight loss commenced at about P70 and progresses rapidly until termination of the trial at P87. In the period from P70 to P87, female and male saline treated *NPC2* ^+/+^ mice gained 7.5% (P|<0.05, 95% CI: 4.0-11.2) and 5.8% (P|<0.05, 95% CI: 3.7-7.9) in weight, respectively (Fig. 3 A and B). During the same time period, age matched saline-treated *NPC2^−/−^* female and male mice experienced a weight loss of 15.4% (P|<0.05, 95% CI: 10.8–19.9) and 16.9% (P|<0.05, 95% CI: 12.2–21.8), respectively. In comparison to the saline treated *NPC2^−/−^* mice, the weight loss observed in NPC2 treated *NPC2^−/−^* mice was significantly reduced and amounted to 6.9% in females (P|<0.05, 95% CI: 3.5–10.3) and 6.4% in males (P|<0.05, 95% CI: 1.0–13.8) during the P70 to P87 period. Our results suggest that NPC2 replacement therapy partially reversed the NPC2 disease induced reduction in body weight. Whether this effect is subscribed to an enhanced organ function and dietary metabolism or the result of improvement of motor and e.g. swallowing function, is beyond the scope of the present report and merits further investigations.

### NPC2 treatment partially corrects visceral cholesterol accumulation in *NPC2^−/−^* mice

**Figure 4:**
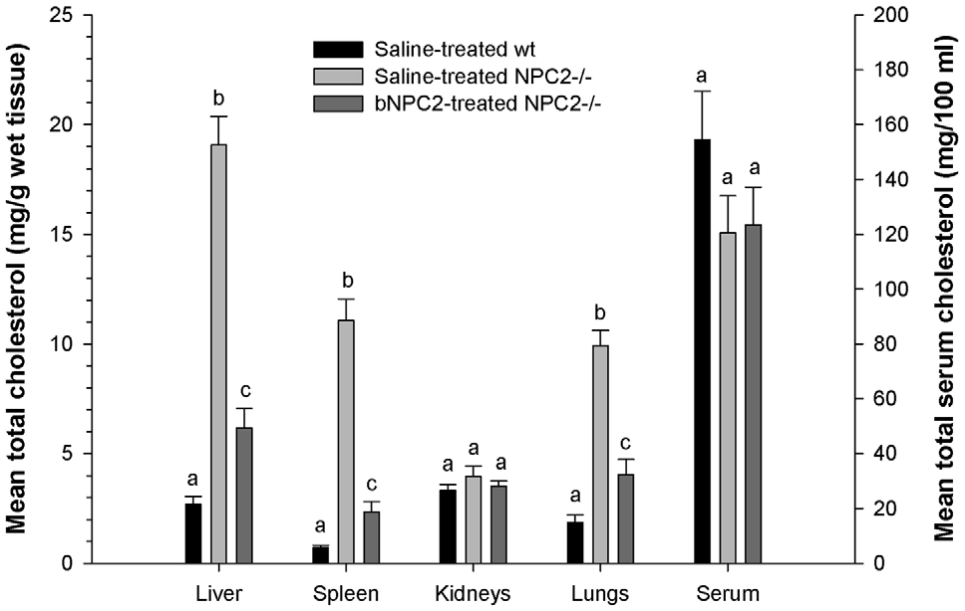
Effect of NPC2 replacement therapy on systemic organs and serum cholesterol storage. Saline treated wild type mice (*black bars*), saline treated *NPC2^−/−^* mice (*light gray bars*), and NPC2 treated *NPC2^−/−^* mice (5 mg/kg) (*dark gray bars*) were injected twice weekly during the evaluation period (P21 to P87). Post mortem total cholesterol concentrations were determined in liver, spleen, kidney, lung, and serum. Each bar represents the mean ± SEM measure for 6 animals in each of the experimental groups done in duplicate. Bars not sharing a letter within a given panel are significantly different (*P*<0.05).

Visceral cholesterol accumulation is a hallmark of NPC disease [1], [8]. Liver cholesterol content was 12.2 fold (P|<0.05, 95% CI: 9.1–16.4) higher in saline treated *NPC2^−/−^* mice compared with age-matched wild type littermates. NPC2-treated *NPC2^−/−^* mice showed a 6.2 fold (P|<0.05, 95% CI: 4.5–8.4) reduction in liver cholesterol levels of that found in the vesicle treated *NPC2^−/−^* mice (Fig. 4). Accumulated storage material was also observed in the spleen of saline treated *NPC2^−/−^* mice where the cholesterol level on averages was 10.3-fold higher than in wild type spleen (P|<0.05, 95% CI: 5.6–18.9). However, the amount of cholesterol entrapped in the spleen of treated *NPC2^−/−^* animals, was reduced by 5.0 fold (P|<0.05, 95% CI: 2.8–8.9) (Fig. 4). Notably, accumulated cholesterol was also reduced efficiently in the lung where the cholesterol level in the saline treated *NPC2^−/−^* mice was 6.8 times (95% CI: 4.3–10.6) higher than detected in wild type mice. NPC2 treatment led to a 2.4 fold reduction (95% CI: 1.5–3.7) in lung sequestered cholesterol compared to the saline treated *NPC2^−/−^* mice (Fig. 4). No significant difference was observed in kidney and serum cholesterol levels among the three experimental groups, although there was a tendency towards lower cholesterol levels in both saline and NPC2 treated *NPC2^−/−^* mice, when compared with the wild type controls (Fig. 4).

### NPC2 treatment abrogates cholesterol storage and visceral histopathology *in vivo*

**Figure 5:**
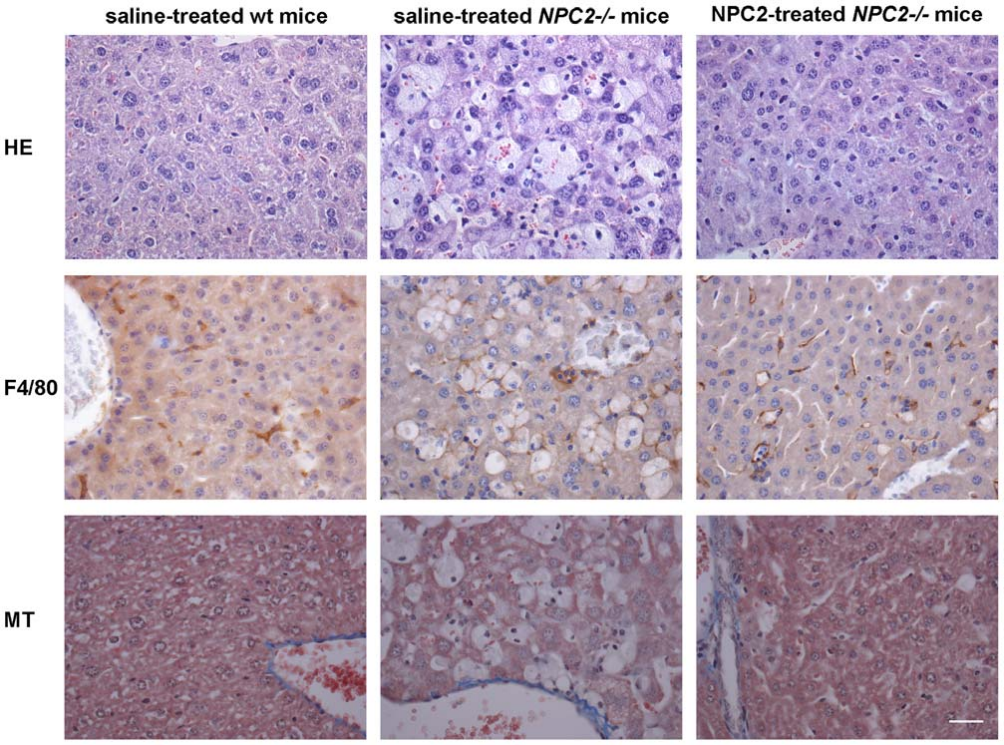
Histochemical and immunohistochemical analysis of NPC2 replacement therapy in murine liver sections. Staining of representative tissue sections of 87 days old saline treated wild type mice (*left panels*), saline treated *NPC2^−/−^* mice (*middle panels*), and NPC2 treated *NPC2^−/−^* mice (*right panels*). Hematoxylin*-*eosin (H&E) staining (*first row*), immunohistochemical localisation of antigen F4/80 positive macrophages (brownish) (*second row*), and Masson's trichrome staining to detect collagen (blue) (*third row*). Lipid laden macrophages (Kupffer cells) are clearly visible and prominent in liver section from saline treated *NPC2^−/−^* mice, whereas only a minority of the macrophages in liver sections from NPC2 treated *NPC2^−/−^* mice are correspondingly loaded. Data are representative of three separate experiments. *n* = 3 animals in each experimental group. Scale bars represent 80 µm.

**Figure 6:**
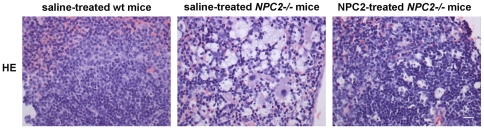
Histochemical analysis of NPC2 replacement therapy in murine spleen sections. Hematoxylin*-*eosin (H&E) staining of spleen from saline treated wild type mice (*left panel*), saline treated *NPC2^−/−^* mice (*middle panel*), and NPC2 treated *NPC2^−/−^* mice (*right panel*). Massive accumulation of lipid droplets was most prominent observed in the spleens of saline treated NPC2*^−/−^* mice. Data are representative of three separate experiments. *n* = 3 animals in each experimental group. Scale bares represent 100 µm.

**Figure 7:**
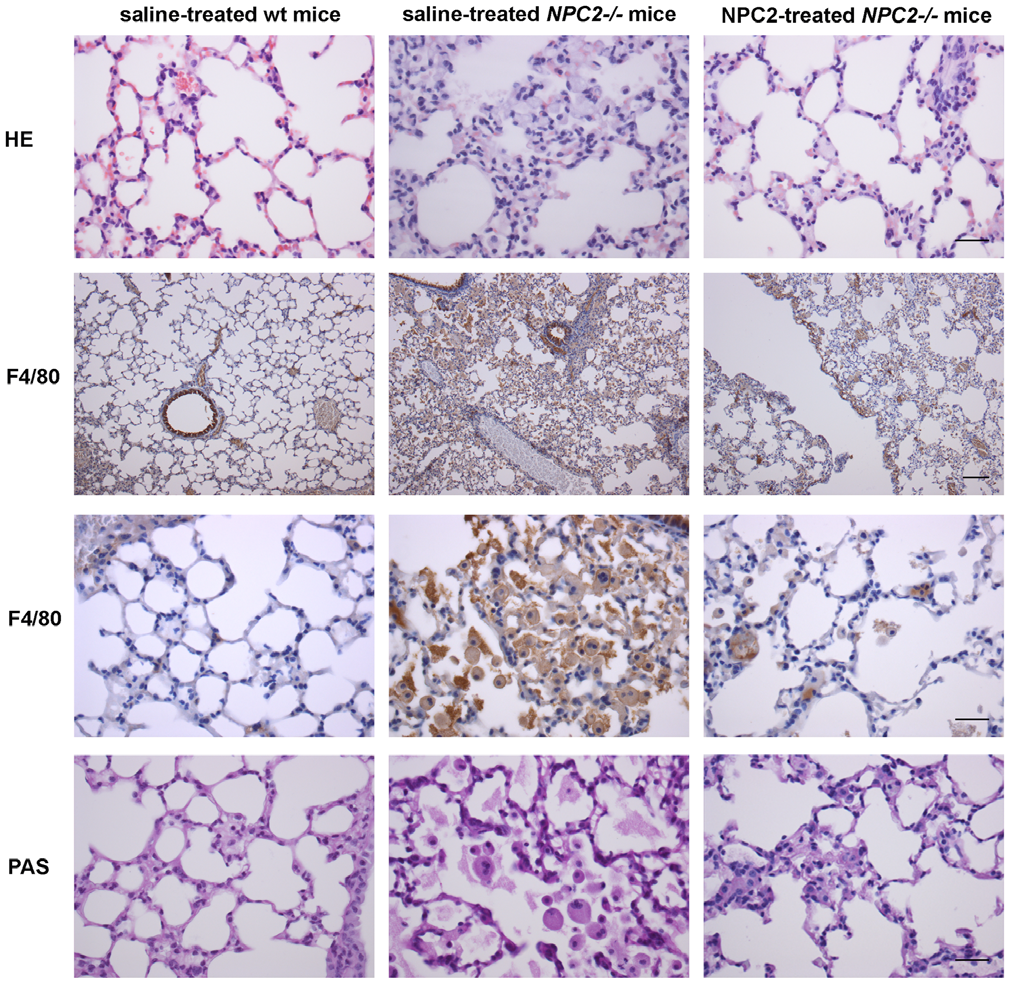
Histochemical and immunohistochemical analysis of NPC2 replacement therapy in murine lung sections. Staining of representative tissue sections from saline treated wild type mice (*left panels*), saline treated *NPC2^−/−^* mice (*middle panels*), and NPC2 treated *NPC2^−/−^* mice (*right panels*). Hematoxylin*-*eosin (H&E) lung staining (*first row*), immunohistochemical localisation of antigen F4*/*80 positive macrophages (brownish) (*second and third row*), and PAS-staining to detect the presence of glycogen (dark purple) (*fourth row*). Data are representative of three separate experiments. *n* = 3 animals in each experimental group. Scale bars represent 30 µm except in second row where it equals 100 µm.

The main visceral histopathological alteration in NPC disease is an accumulation of lipid-laden foam cells in the visceral organs [28], [29], [30], [31]. Accordingly, hepatic H&E- and immunohistochemical-staining with a macrophage specific antibody (F4/80) revealed large numbers of sinusoidal lipid-laden Kupffer cell clusters in tissue sections from saline treated *NPC2^−/−^* mice. In contrast, the histological changes were minimal in liver sections from NPC2 treated *NPC2^−/−^* mice, which showed no significant lipid accumulation or gross pathological changes and instead appeared much similar to liver sections from age-matched wild type mice (Fig. 5). Masson-Trichrome staining revealed perivascular collagen deposition in both *NPC2^−/−^* mice and wild type littermates. However, there was no obvious fibrosis in any of the groups. The histopathological findings of the liver sections from saline treated *NPC2^−/−^* mice are consistent with previous studies of liver histopathology in *NPC1^−/−^* mice [30], [31]. Similar to the liver, H&E staining of spleen tissue sections revealed a marked NPC2-dependent reduction in histiocyte infiltration (Fig. 6). Saline treated *NPC2^−/−^* mice had intra-alveolar and interstitial accumulation of foamy macrophages and intra-alveolar accumulation of Periodic Acid-Schiff (PAS) positive material (Fig. 7), consistent with alveolar lipoproteinosis. Again, NPC2 treatment led to reduced macrophages infiltration, a reduction in PAS-positive material, and an overall improvement in alveolar architecture.

### NPC2 treatment has insufficient effect on cholesterol storages in affected regions of the brain

**Figure 8:**
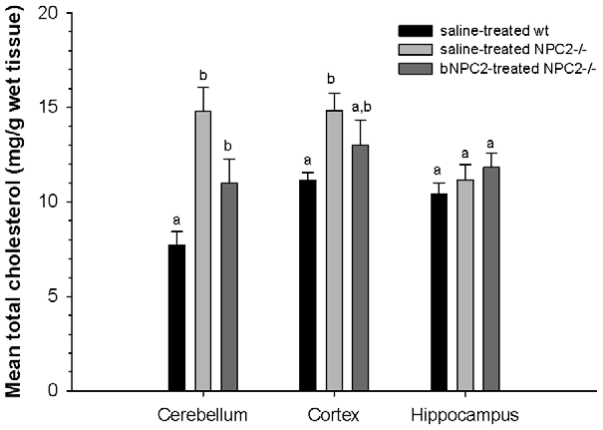
Effect of NPC2 replacement therapy on murine brain cholesterol storage. Total cholesterol levels in cerebellum, cortex, and hippocampus was measured postmortem in saline treated wild type mice (*black bars*), saline treated *NPC2^−/−^* mice (*light gray bars*), and NPC2 treated *NPC2^−/−^* mice (*dark gray*). Each bar represents the mean ± SEM for 6 animals in each of the three groups. Bars not sharing a letter within a given panel are significantly different (*P*<0.05).

Compared to the visceral organs, a less dramatic but still significant increase in cholesterol levels occurred in specific brain regions from both saline and NPC2 treated *NPC2^−/−^* mice. This was most notably the case in cerebellum and to a lesser extend in cerebral cortex. Contrary to this, no difference was found in the levels of hippocampal cholesterol comparing the *NPC2^−/−^* and wild type experimental groups (Fig. 8). Although not statically significant at the 5% level, we noticed that the cerebellar cholesterol load tended to decrease in the NPC2 treated *NPC2^−/−^* mice (1.4 fold above wild type, 95% CI: 1.1–1.9) compared to their saline treated hypomorph littermates (1.9 fold above wild type, 95% CI: 1.4-2.7). The same tendency towards a reduction in sequestered cholesterol was not observed in cortex (Fig. 8). Our results are compatible with no effect or, at best, a limited effect of NPC2 on cholesterol deposition in the brain under the chosen experimental conditions.

### NPC2 treatment does not influence behavioral and neurohistopathological abnormalities in *NPC2^−/−^* mice

Homozygous NPC2-targeted mice from heterozygote mating were born at numbers below the predicted Mendelian frequency (n = 65 for wild type, n = 139 for heterozygous, n = 36 for NPC2-targeted mice). The coat condition and mean body weight of both female and male NPC2^−/−^ mice entering the replacement trial at p21 were similar to gender-matched wild-type mice. A progressive decrease in relative weight gain commenced over the following two weeks among the gene-targeted mice. However, they presented no outward neurological signs of disease until eight weeks of age when tremor and ataxic gait became detectable. The motor symptoms became progressively more severe as the disease approached end stage and euthanasia was performed.

**Figure 9:**
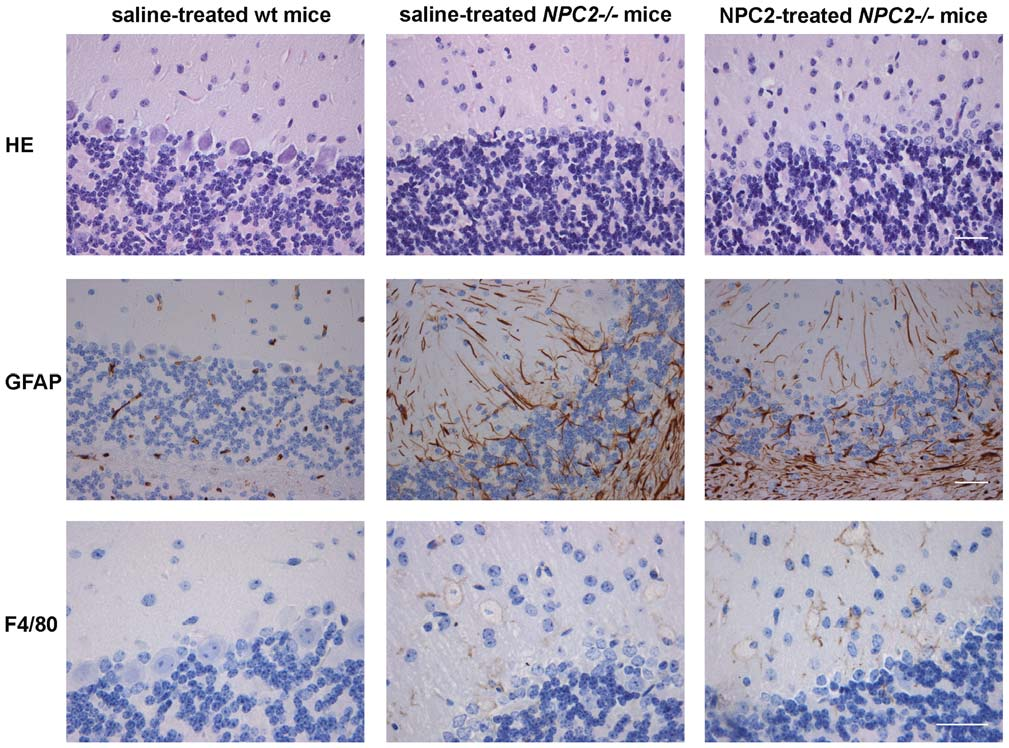
Histochemical and immunohistochemical staining of cerebellum. Staining of representative cerebellum sections from saline-treated wild type mice (*left panels*), saline treated *NPC2^−/−^* mice (*middle panels*), and NPC2 treated *NPC2^−/−^* mice (*right panels*). Hematoxylin*-*eosin (H&E) staining (*first* row), immunohistochemical GFAP-staining for detection of astrocytes (brownish) (*second row*), and immunohistochemical localisation of antigen F4*/*80 positive macrophages (brownish) (*third row*). Data are representative of three separate experiments. *n* = 3 animals in each experimental group. Scale bars represent 30 µm.

Intravenous NPC2 treatment had no noticeable effect on gross neurological symptoms as also the NPC2 treated *NPC2* ^-/-^ mice began to display tremor and a pronounced ataxic gait at about P60. These symptoms worsened progressively until euthanasia at P87. In accordance, H&E-staining of the cerebellar cortex revealed massive loss of Purkinje cell in both saline and NPC2 treated *NPC2^−/−^* mice (Fig. 9). Furthermore, marked astrocytosis adjacent to degenerative lesions was shown by glial fibrillary acidic protein (GFAP) immunoreactivity (Fig. 9). Several large pale cells with vacuolar cytoplasm were observed, which stained positive with anti-F4/80 IgG. The antigenicity and morphology were consistent with the occurrence of microglia and their numbers were not significantly influenced by the NPC2 treatment.

## Discussion

The present study is the first to report the effect of intravenous NPC2 replacement therapy in a mouse model of NPC2 disease. Low amounts of potentially NPC2 neutralizing antibodies were detected in some of the NPC2 treated animals, which may have had at least marginal influence on the efficacy of the cure. However, no indication of anaphylaxis and no loss of animals were observed during the trial.

The major organ sites of pathology in NPC disease are the liver, spleen and lung, as well as the brain. Following termination of the trial, the group receiving NPC2 therapy had pronounced lower cholesterol levels in the liver, spleen, and lung than saline treated, age-matched *NPC2^−/−^* control animals. Histological analysis corroborated the biochemical results and showed a clear reduction in the number of fat-laden cells and disease-associated macrophage infiltration of the NPC2 treated *NPC2^−/−^* group. We did not find any significant variation in kidney, whole brain and serum cholesterol comparing *NPC2^−/−^* with wild type mice. However, measured separately, cerebellum and to a lesser extent cerebral cortex derived from *NPC2^−/−^* mice revealed a statistically significant increase in the proportion of cholesterol. Although we noticed a tendency towards a cerebellar cholesterol reduction in the NPC2 treated animals, all of the *NPC2^−/−^* mice developed severe ataxia by the end of the treatment schedule. Histological analysis again corroborated, by showing the same degree of cerebellar Purkinje cells loss and astrocytosis in both NPC2 treated and saline treated *NPC2^−/−^* mice.

It is of interest to compare our NPC2 replacement results to other intervention studies of animal models of NPC disease. Most striking are data demonstrating that the lipid chelator 2-hydroxypropyl-β-cyclodextrin (CD) partially can replace the function of NPC1 and NPC2 proteins [32], diminishes neuropathy, delays motor deficits, and increase median survival in both *NPC1^−/−^* and *NPC2^−/−^* mice [13], [14], [33], [34]. Besides the CNS effects, the systemic response to CD seems comparable to NPC2 replacement therapy, except in the lungs where substantial cellular cholesterol accumulation and increasing macrophage infiltration has been observed [33]. As a result, CD treated mutant mice developed a pulmonary alveolar proteinosis that appeared similar to that seen in lung biopsy specimens taken from NPC2 deficient infants [28], [29], [35]. The reason for the unresponsiveness of the lungs towards CD is unclear, as is the mechanism by which CD bypasses the action of NPC1 and NPC2. However, recent data indicate that CD works from inside late endosomes/lysosomes compartment, suggesting that endocytic uptake is significant for CD potency [32]. Accumulation of functionally inactive, cholesterol rich surfactant has been proposed to be the mechanism underlying the respiratory symptoms in NPC2 disease patients [29]. Patient's bronchoalveolar lavage fluid reveals accumulation of enlarged foamy and vacuolated alveolar macrophages embedded in the surfactant, in keeping with their innate inability to mobilize phagocytosed cholesterol. The implication may be that in the affected lung environment, receptor-mediated endocytosis of NPC2 is more efficient than fluid-phase CD pinocytosis in enabling the macrophages to acquire the macromolecules they needed in order to ameliorate pulmonary pathology. Alternatively, NPC2 may have other biological functions, which influence the phenotypic diversity observed in NPC2 and CD treated lung. Consistent with this notion, it has recently been reported that NPC2 deficiency in human fibroblasts confers their activation [36]. Intriguingly, autopsy has revealed that NPC2 deficiency is associated with pronounced pulmonary fibrosis [37], known to be primarily mediated by activated fibroblasts [38]. Because normalization of cholesterol in the *NPC2^−/−^* fibroblasts failed to correct their activated phenotype [36], it is possible that the ameliorating effect experienced by NPC2 therapy is independent of its function in intracellular cholesterol trafficking. The latter is in line with new and interesting studies, highlighting NPC2 as a potentially multi-functional protein involved in a number of regulatory processes, including hematopoiesis [39], immunity [40], somatic cell plasticity and adipogenesis [36], [41], and in papillae formation [42], all apparently independent of NPC2 cholesterol binding ability. Furthermore, a positive correlation between extracellular NPC2 and cholesterol biliary secretion has been reported [43]. Therefore, a well-grounded decision on the basis of the alleviating effects of NPC2 replacement has to await further investigation into the cholesterol transport dependent and independent activity of the protein.

Although the majority of data implicate cholesterol accumulation as the primary defect in NPC disease, biochemical analyses of lipid extract from human and murine NPC brains have revealed increased levels of glycolipids [44]. Thus, a second therapeutic agent successfully tested in animal models of NPC disease is miglustat (N-butyl-deoxynojirimycin, Zavesca®), an imino sugar-based inhibitor of the glycosphingolipid biosynthetic pathway [45]. Animal models responded positively to treatment, resulting in reduced ganglioside accumulation, delayed onset of neurological symptoms, increased Purkinje cell survival, and extended animal life span [46]. In Europe, miglustat, has recently been approved for specific treatment of the neurological manifestations in NPC disease and clinical trials have demonstrated a mild clinical neurological improvement or stabilization, but did not indicate clinically relevant systemic organ effects, where cholesterol accumulation was almost unaffected [11], [12], [47]. Considering NPC2 replacement therapy ameliorates the lung and visceral complications experienced by our animal model of NPC2 disease as well as affected patients, we submit that a combination of NPC2 and CD and/or miglustat may represent a highly attractive novel therapeutic intervention for the handful of patients suffering from NPC2 disease.

The NPC2 treatment led to no obvious improvements in CNS disease. This is likely due to the inability of the injected NPC2 protein to cross the blood brain barrier (BBB), which restricts the passage of proteins into and out of the brain parenchyma [48], [49]. The NPC2 replacement therapy was initiated three weeks after birth, which may have reduced its therapeutic potential in the CNS. This is underlined by prior preclinical studies in mouse models showing M6P-containing enzymes cross the neonatal BBB through a developmentally regulated M6P-receptor-mediated transport mechanism [50]. As the M6P-receptor-dependent transport across the BBB in mice is discontinued after about two weeks postpartum [51], the fact that NPC2 therapy with the M6P-tagged NPC2 [4] did not stall CNS degeneration may relate to the onset of the treatment. Two strategies have previously been used to overcome the decreased expression of M6P-receptor; 1) high dose replacement therapy, e.g. in a mouse model of adult mucopolysaccharidosis type VII [52], and 2) induction of M6P-receptor expression using epinephrine which appears to restore transport kinetics to the postnatal state [53]. Whether adoption of such strategies would have any impact on the manifestations of the neurodegenerative process is an important question that needs to be answered by further research. Notable, although we have found that milk derived NPC2 is able to bind the cation-independent M6P-receptor, the rate of M6P-receptor internalization vary depending on the mannose phosphorylation status of the ligand. As the intrinsic affinity of NPC2 for the M6P-receptors is unknown, our current data do not allow us to conclude whether the NPC2 is transported to lysosomes via the M6P-receptor, in an M6P-independent manner mediated by alternative receptors, or by fluid-phase pinocytosis. Regardless, an alternative delivery strategy is to bypass the BBB by direct injections into the cerebral circulation, which previously has been exploited successfully in other models of neurodegenerative lysosomal storages diseases [54], [55].

Minimal changes in whole brain cholesterol have been reported in *NPC1^−/−^* mice and previous studies showed that cholesterol levels in brains are similar to, if not lower, compared with levels observed in wild type mice [56], [57]. We extended previous investigations on whole *NPC1^−/−^* brain by investigating the cholesterol levels in three different regions of the *NPC2^−/−^* brain (cerebellum, cortex, and hippocampus). These data revealed that the cholesterol level of hippocampus is similar among NPC2 deficient- and wild type mice. There was a marginal increase in the cholesterol pool of the cortex in *NPC2^−/−^* mice, whereas cerebellum revealed a more clear-cut cholesterol excess compared to wild-type brains. Recently, Bi and coworkers reported that cholesterol accumulation resulting from lack of functional NPC1 protein occurs not only in neurons but also in microglia [58]. These observations are in line with our results as microglia infiltration was observed in cerebellum of *NPC2^−/−^* mice. Whether glial activation represents a reactive response to neuronal damage, or results from intrinsic alterations due to NPC2 deficiency remains undetermined. The finding that cholesterol is significant elevated in cerebellum may appear in discord with previous reports based on *NPC1^−/−^* whole brain homogenates. However, considering the weight of the adult mouse cerebellum constitutes ∼10% of whole brain weight, it may not be surprising that the mean increase of total cholesterol in this region of the brain do not significantly contribute to the overall measurement of cholesterol in the whole brain. In support of this, progressive accumulation of cholesterol as detected by staining with the fluorescent dye filipin has recently been observed in cerebellum sections from in *NPC1^−/−^* mice [59]. The targeted rescue of the visceral organs in the *NPC2^−/−^* mouse allowed us to observe the benefits systemic improvements alone could have on neurodegeneration NPC disease. Based on our data it seems likely that the neurodegenerative effects observed is a direct effect of NPC2 deficiency and not a secondary metabolic side effect in response to e.g. liver damage.

### Conclusion

We present data from preclinical studies that regular intravenous administrations of NPC2 ameliorate many of the visceral changes caused by loss of functional NPC2 protein. Thus, systemic delivery of NPC2 seems a promising option for treating the non-cerebral complications in NPC2 disease, which may be readily translatable to the clinical setting. In particular, this therapeutic approach may be suitable for patients who experience chronic respiratory and liver failure in early infancy for which there is currently no adequate therapeutic approach. It remains to be investigated whether improved efficiency of administered NPC2 to relieve the cerebral symptoms can be gained by changes in route of administration, dose level, or therapy onset. The two latter have previously been shown to influence survival time in CD treated *NPC1^−/−^* mice [14]. Furthermore, intrathecal administration of CD in a feline model harboring a missense mutation in *NPC1* has recently been shown to better ameliorate aspects of the neurological disease and extend lifespan when compared to systemic injection [60]. It remains to be seen if the intrathecal applicable strategy is a potential avenue for NPC2 replacement in CNS as well.

## Acknowledgments

Mice heterozygous for the NPC2 mutation were kindly supplied by the Wadsworth Center Transgenic and Gene Knockout Core Facility, Albany, NY, USA. We thank Margit Skriver Rasmussen, Jette Krüger Jensen, and Karen Lykkegaard Christensen for expert technical assistance.
